# The Association between Active Mobility and Subjective Wellbeing during COVID-19 in MENA Countries

**DOI:** 10.3390/healthcare10091603

**Published:** 2022-08-23

**Authors:** Behzad Ranjbarnia, Mohammad Javad Kamelifar, Houshmand Masoumi

**Affiliations:** 1 Department of Geography and Urban Planning, Faculty of Planning and Environmental Sciences, University of Tabriz, Tabriz 5166616471, Iran; 2 Tabriz Metropolis Municipality, Tabriz 5174643119, Iran; 3 Center for Technology and Society, Technische Universität Berlin, Kaiserin-Augusta-Alle 104, 10553 Berlin, Germany; 4 Department of Transport and Supply Chain Management, College of Business and Economics, University of Johannesburg, Johannesburg 2006, South Africa

**Keywords:** active lifestyle, subjective wellbeing, ordinal logistic regression, COVID-19, Middle East and North Africa

## Abstract

Objective: To augment the international scientific approach to raising public mental health through active lifestyle among adults, we added the evidence of the association between physical activity and subjective wellbeing in the Middle East and North Africa region by emphasizing the mediator—COVID-19. This study aimed to identify the correlations between active mobility and subjective wellbeing during the COVID-19 pandemic in a sample from Tabriz, Iran, which has not yet been tackled in previous study. Methods: We finalized an online survey (N = 603) from adults between 5 June and 15 July 2021. This group reported their individual and socio-economic characteristics and their perception features and location (e.g., home, work) during COVID-19. The paper developed three ordinal logistic regression (OLR) models to examine the association between active mobility types such as commute, non-commute, frequency of active travel to parks and services per week, and different subjective wellbeing including: 1- life satisfaction, 2- feeling energetic, and 3- peaceful mind while controlling for socio-economic variables (e.g., age, gender, education, job, and income) and objective commute distance. We also incorporated the most relevant objective (street length, land-use mix, number of intersections, and building and population density) and subjective (perceived distance to different services, perceived walking places, and perceived facilities attractiveness) factors. Results: Positive response relationships between four types of physical activity levels and subjective wellbeing scores were demonstrated in all of the three developed models (with significant levels of 0.05, and 0.1) with appropriate model fits, which confirmed the existing literature. However, these relationships showed different patterns (varied significant levels) for each type of subjective wellbeing. In addition, the factors including street length (*p* value: 0.004), perceived walkable places (*p* value: 0.021, 0.068, 0.017, and 0.025) (positively), population density, and perceived distance to shopping malls (*p* value: 0.076, <0.0001, and 0.059 (negatively) were associated with different indicators of subjective wellbeing. Conclusion: As for the implication of our study, special actions by urban authorities such as increasing mixed-use and creating attractive places will be required to enhance the walkability of the neighborhoods. Moreover, notifying the adults regarding the benefits of physical activity is much more needed.

## 1. Introduction

The COVID-19 pandemic has infected and harmed a large number of individuals around the world [1,2]. Because of the disease’s ability to spread from person to person [3], many countries imposed mobility restrictions and required individuals to stay at home unless providing essential services, working in a vital job, attending to medical problems, or engaging in one form of exercise, e.g., running, walking, or cycling, and so on [4]. These evidence altered the lifestyle of most citizens, meaning that many individuals had to rethink their travel choices. This group faces three broad options: changing to walking or cycling (active travel), staying at home, or using a car or motorcycle [5].

It has been said that such a lifestyle (e.g., the physical inactivity) resulting from the pandemic has caused various detrimental impacts on human health and wellbeing. For example, sleep difficulties and an increase in comfort eating are believed to have resulted during the pandemic, leading to a loss of weight control [6]. According to previous studies, the pandemic has hindered individuals from associating with others and is likely to have harmed emotions of self-esteem and circumstances [7], stress [8], and depression [9]. These researches have illustrated that in individuals who are usually regularly active, even a small period such as one week of inactivity is sufficient to considerably enhance negative feelings [10], which indicates that the general subjective wellbeing (SWB) level is considered low during COVID-19 [11]. In turn, physically active travel options allow individuals to include physical activity (Based on WHO (2020); physical activity refers to all movement including during leisure time, for transport to get to and from places, or as part of a person’s work.) into their daily routines, and a comprehensive evaluation of research suggests that they are beneficial to their health. Individuals who cycle to work, for example, have a lower chance of death from any cause; individuals walking to a job place have a lower risk of cancer; and individuals who switch from driving to active modes have a lower body mass index (BMI). Moreover, the latest studies show that regular and maintained participation in physical activity is associated with positive mental health [12] such as mental wellbeing [13], happiness [14], and life satisfaction [15,16,17,18,19,20]. These positive correlations were found in youths [21], adults [22], and the elderly [23].

The existing literature evaluated the relationship between active transport (physical activity) and subjective wellbeing. The previous words provide evidence for positive correlations between active transport and subjective wellbeing [24,25]. The majority of these studies have emphasized the leisure or recreation walking and cycling [26,27,28], and a few of them have addressed the impact of active job commute and regular physical activity on mental wellbeing [29,30]. However, for all we know, no study has comprehensively targeted investigating the impact of various modes of active transport on subjective wellbeing. In other words, in this paper, the authors incorporated different types of active mobility and physical activity which is unprecedented in the previous works. In addition, the current research was carried out in the COVID-19 era, suggesting its direct or indirect impacts on the variables of this study. Ultimately, the research targeted a sample from large cities of MENA which adds on the uniqueness of this research. Thus, this research is intended to address the mentioned gaps by presenting a holistic measure. In other words, the research aims to analyze the association between different active mobility types (active commute, non-commute, and frequency of active travel to services and parks), and subjective wellbeing indicators (life satisfaction, feeling energetic, and peaceful mind). This objective will be even more enriched by incorporating the role of both objective and subjective environmental factors. Furthermore, the research on the effects of the pandemic on the changed patterns of association between active transport and subjective wellbeing can be considered a global public health need. Such need is also more demanding in developing countries than in developed (A country which has an effective rate of industrialization and income personally is known as Developed Country. A developing nation is a country that has a slow rate of industrialization and low per capita income. Unemployment and Poverty (“Difference Between Developed Countries and Developing Countries, Available online: https://keydifferences.com/difference-between-developed-countries-and-developing-countries.html (accessed on 17 April 2020))” ones due to insufficient health data, infrastructures, and relevant studies.

Hence, examining the association between different kinds of physical activity and wellbeing during the pandemic may promote measures which target increasing active modes of transport, which ultimately will lead to a growth in wellbeing and health among individuals. As a matter of fact, this research will make an essence for better understanding of the association among active mobility and subjective wellbeing in a large city of MENA countries for scholars, urban authorities, and individuals in a comprehensive manner which finally will contribute to the increase in health and wellbeing. This article has taken four modes of active travel, including commute, non-commute, and active travel frequencies to service and parks, as independent variables whose impact will be assessed on the three dependent variables of subjective wellbeing, including life satisfaction, feeling energetic, and peaceful mind. All of these measures will result in three output models. This approach will aid planners and authorities to have a comprehensive and accurate understanding of the active lifestyle impact on subjective wellbeing in large cities of developing countries. Thus, objectives of the study are: 1—to recognize the effects of active transport behavior of adults on the subjective wellbeing in the large cities of developing countries during COVID-19; 2—to compare the output models driven from the impact of active modes of walking on different dependent variables of subjective wellbeing during COVID-19.

The following units make up the rest of the article. Section 2 examines the existing research on active travel behavior, subjective wellbeing, and their relationship during the COVID-19 pandemic, as well as the state of similar studies in developing countries. The approaches utilized in this paper are discussed in Section 3. Following that, in Section 4, we present a summary of the statistical approaches’ outcomes. Section 5 discusses the models’ parallels and variations in correlations between an active lifestyle and subjective wellbeing during the COVID-19 pandemic, as well as comparisons to studies conducted before and after the outbreak. Finally, the conclusion is debated in Section 6.

### 1.1. Literature Review

#### 1.1.1. Active Transport and Subjective Wellbeing

The connection between active transport and physical and mental health has been investigated by many researchers [31,32]. Most of these studies have put their emphasis on physical health. For instance, the levels of active transport have been correlated with more regular levels of moderate-to-vigorous activity [33], lower body mass index, obesity risk [34,35,36,37], reduced cardiovascular disease (CVD) risk [30,33,38], and diabetes [38]. However, less is examined about the correlations between active lifestyle and subjective wellbeing and quality of life. Most studies to date have been limited in the number of outcomes evaluated.

Researchers have tried to understand the nature, determinants, and consequences of subjective wellbeing with the rise of new courses such as hedonic psychology, positive psychology, and happiness economics [35,36]. Subjective wellbeing (SWB) is defined as a multidimensional concept that refers to individuals’ levels of wellbeing, general satisfaction with their lives and essential life domains, and their associated emotional states [37]. This concept has recently become a topic of interest within the transportation research community related to studying the pleasure of traveling [28], travel stress, and travel happiness and satisfaction [39]. When compared to vehicle journeys, walking has been correlated with higher life satisfaction [40], while biking commutes has been connected with lower risk of being stressed [41] as well as lower illness absence and better and higher subjective wellbeing [42]. The majority of the studies have confirmed that active travel is considered beneficial in supporting behaviors that improve health and wellbeing, reinforcing regular physical activity participation and subsequently aiding positive subjective wellbeing [43]. For example, Avila-Palencia et al. (2017) have found that cycling has more weight on enhancing subjective wellbeing than walking [42].

Yet, there remain critical gaps in the evidence regarding the associations between active commuting and wellbeing [28]. In most studies, such association has been investigated regardless of the comprehensive research of active travel purposes. These studies have neglected to separate various active lifestyle targets. Only a few researchers have measured whether there are variations between active lifestyle domains and their impact on effective wellbeing. Physical activity in recreation or leisure time seems to have a more positive effect on wellbeing than active journeys to work or transport-related variables [29]. These variations in the effects of different forms of physical activity may result from the complicated level of perceived independence and self-determination, which are regularly correlated with leisure-time physical activities [14]. Another research indicated that walking and cycling travel was associated with enhanced subjective wellbeing in physically, vigorously, and moderately active entities [44]. Such complicated nature of the association between perception and active lifestyle calls for a comprehensive analysis of emotions related with varied types of active lifestyles. Thus, the innovation in this study is that we target and compare the various types of active mobility (commute, non-job commute, frequency of active commuting to services, and parks) and their impact on different aspects of subjective wellbeing, including life satisfaction, feeling energetic, and peaceful mind.

This study aims to examine these associations by considering the mediator of the COVID-19 outbreak. There are even apparent knowledge gaps in understanding the role of the pandemic on the associations between various types of active travel and subjective wellbeing.

#### 1.1.2. Situation during COVID-19

To cope with the pandemic’s physiological impact and restore homeostasis, self-care has been highlighted as increasingly important [7], and physical activity (PA) is a form of self-care. In the context of key physiological needs that are disrupted by the pandemic [7,45], an active lifestyle, as mentioned, has previously been shown to affect subjective and objective wellbeing [46,47,48,49] According to recent studies, mental health was at an all-time low during the pandemic [11,47]. Banna et al. (2022), for example, surveyed individuals and discovered that anxiety and depression symptoms were present in 33.7 percent and 57.9 percent of those surveyed, respectively [48]. A similar finding was made in the United Kingdom [49]. Because regular and sustained engagement in an active lifestyle is connected with beneficial health outcomes such as mental wellbeing [13], happiness [14], and life satisfaction [16,21,22], this decline in mental health is most likely due to mild declines in physical activity [50]. Physical exercise, as a preventive factor against poor mental health, is likely to play an important role in the pandemic [46,51].

During the COVID-19 crisis, evidence regarding the link between physical activity and mental health is still being gathered. Despite this research, it has yet to be empirically studied whether different types of active travel, such as daily commutes to work or leisure active travel, contribute to effective wellbeing in different ways during the pandemic. No study has holistically investigated the relationship between active travel and various domains of subjective wellbeing during confinement, such as the quarantine caused by COVID-19. Thus, information on the effect of lockdown strategies from multiple global regions may help governments improve future lockdown strategies to minimize or mitigate adverse physical and mental health effects. Accordingly, we aim to explore the relationship between different types of active travel (job-commute, utilitarian, and leisure) and various dimensions of subjective wellbeing (quality of life, being energetic, etc.) among adults during the COVID-19 pandemic. It may help the community members to improve their subjective wellbeing through active travel in the face of a future pandemic or potential waves/relapses.

#### 1.1.3. Condition in Developing Countries

In contextual studies, inconsistencies are observed between developing and developed nations. Active travel’s health impacts vary slightly across industrialized and poor countries. Developed countries have a more elevated economic level, and more individuals engage in physical activity [52]. Furthermore, exercise has a greater favorable impact in industrialized countries. In contrast, emerging countries are experiencing a rapid economic expansion, which has resulted in increased pollution and rapid urbanization, resulting in reduced levels of physical activity among the population [50]. Many individuals have not yet established new healthy lifestyles as a result of the transition from traditional to modern cultures, such as daily or weekly physical exercise, which has resulted in disparities in the influence of physical activity on health across countries.

There are fundamental research gaps in contextual fields concerning the relationship between active lifestyle and emotional health. As a result, in a rapidly urbanizing region such as MENA and a country such as Iran, individuals’ living circumstances, physical exercise habits, and mental health are changing. Furthermore, earlier research has demonstrated a relationship between walking (not cycling nor a combined factor) and mental health [53]. However, these studies have emphasized only one type of walking and the outcome variable of wellbeing. This research will explore the link between various types of active lifestyle and different domains of subjective wellbeing to address such gaps during the COVID-19 pandemic in Tabriz, Iran. This research will aid in a better understanding of the relationship between active mobility and emotional health in the MENA region, as mediated by COVID-19. This case study of Iran will also aid in a better understanding of the growing “lifestyle–environmental health” challenges in other emerging countries. The outcomes of this study could contribute to the increase in active mobility and physical activity levels which will lead to better results in the individuals’ subjective wellbeing and health. Moreover, the results can act as a great facilitator in enhancing the awareness of urban authorities and citizens about the importance of active mobility in the society.

This paper intends to address the following questions to predict the subjective wellbeing through active mobility habits in the understudied context of MENA:

(1). What associations exist among the various types of active mobility/physical activity and different domains of subjective wellbeing during COVID-19 in the large cities of the MENA region?
(2). What similarities and differences exist among the three patterns resulting from the impact of active lifestyle on the different variables of subjective wellbeing?

We hypothesize the existence of positive correlations between all kinds of active lifestyle (including commute, non-commute, and frequency of active trips to parks and services per week) and subjective wellbeing (including life satisfaction, feeling energetic, and peaceful mind) during the COVID-19 pandemic. However, we hypothesize that distinct patterns do exist resulting from the impact of various variables of active lifestyle on different measures of subjective wellbeing. Moreover, this paper assumes that the street length as a mediating factor has a positive role in predicting the association between active lifestyle and subjective wellbeing. However, the factor of population density as another mediating variable negatively correlates with subjective wellbeing during COVID-19 in the metropolitan cities of the MENA region.

## 2. Materials and Methods

### 2.1. Case Study

Due to the high similarities in socio-cultural features and urban form (e.g., monocentric urban structure, poor public transport which increases car dependency) affecting attitudes toward the travel behavior of inhabitants in large cities of the MENA region (The results of this study can be generalized for large cities in MENA.), the finding can potentially be generalized [54], Thus, Tabriz has been chosen as a case study, which can be a good context in Iranian cities as an example of emerging markets in the MENA region.

Tabriz is a metropolitan city which is located in northwest Iran with approximately 1.6 million citizens (2022). This town is considered as one of the modern industrialized Iranian cities. Similar to any other large city in Iran, this town has witnessed swift transformation during the last few years. Moreover, renovations in urbanization after the appearance of modernism in planning have demolished the conventional elements and constructions of the city.

Furthermore, the organic system and functional diversity of cities and urban communities have been supplanted by functional zoning. As a result, walkable neighborhoods have changed, and the city’s overall walkability has declined substantially. To put it another way, the bus rapid transit (BRT) system was established roughly 15 years ago to alleviate the high traffic on the city’s major boulevards, and one of the metro routes recently began servicing inhabitants. However, this city suffers from a scarcity of public transport systems, shifting many citizens to use their cars on their journeys. Additionally, non-supportive environmental factors, including lack of perceived social support, poor political and legal support, climate conditions, air pollution, seasonal limitations, and recent restrictions of the COVID-19 pandemic, are the most substantial barriers to physical activity in Tabriz [55]. Despite these barriers, the city was selected as the first hygienic and healthy city in Iran in 2020 [56].

### 2.2. Data and Variables

This research is based on secondary data from geographic information system (GIS) layers and data from an online questionnaire survey conducted between 5 June and mid-July 2021. This time coincided with general restrictions imposed by Iran’s government (In the time of sampling, no strict lockdown was introduced by the government, because there was no disease wave at the time.), and the sampling was performed by asking about the current situation of the respondents. Considering the interdisciplinary nature of this study, two different types of questionnaires (NEWS (Neighborhood Environment Walkability Scale) for neighborhood walkability, and PGWBI (Psychological General Wellbeing Index) for subjective wellbeing) were used. The selection of respondents was based on an online random sampling method among the citizens of Tabriz (with diverse socioeconomic background from different urban contexts). The information regarding the respondents was given by the respondents about their socio-economics, their perceptions of distance to land uses and services and their neighborhood walkability, mobility mode choice for commute and non-commute trips, physical activity (walking to green spaces and services), and their subjective wellbeing (feeling of life satisfaction, feeling of being energetic, and peaceful mind). Only 670 of the 1091 citizens who began responding to the questionnaires completed enough questions to be used in the analyses. Due to item non-response, some samples were removed from the modeling procedure (603 remained). According to Cochran, the total sample size was 603 and is representative of Tabriz (1963). This report is simply an exploratory study aimed at identifying the link between active mobility, physical activity, and residents’ subjective well-being during the COVID-19 outbreak in large Iranian cities as a model for large MENA cities.

The employed indicators including individual features, travel mode choice, respondents’ perception regarding neighborhood walkability, subjective wellbeing, and built environment (physical) indicators are depicted in Table 1. Binary, continuous, categorical, and ordinal variables are used to classify the variables. Age, income, job type, commute distance, land use mix, distance to public transportation, and population and building density are all continuous factors. In addition, access to various services and land uses, facilities attractiveness, perceived walkable places, and subjective wellbeing factors are all categorical characteristics.

### 2.3. Analysis Methods

Three ordinal logistic regression (OLR) models were generated. For this aim, these models were obtained using SPSS. 26 software package. Accordingly, the variables such as mobility mode choices (active and non-active types of trips for commute, non-commute, green spaces, and services), socio-economic (control factors), and subjective and objective walkability were employed as the independent variables to measure the status of subjective wellbeing as the dependent variable. Next, we developed three OLR regression models for three sub-criteria of subjective wellbeing (namely feeling of life satisfaction, feeling of being energetic, and feeling of being relaxed/peaceful) during the outbreak of COVID-19. The obtained models showed how active lifestyle affects the subjective wellbeing of residents during the pandemic. Through the three models, the status of citizens’ subjective wellbeing was analyzed based on their mobility type and their built environment and the residents’ subjective perceptions (objective). As independent variables, the first set of OL models used 26 variables employing backward approach. Unlike the forward approach, the backward approach avoids producing so-called suppressor effects. (These suppressor effects occur when predictors are only significant when another predictor is held constant). Based on the highest *p*-value, insignificant variables were removed from the OLR models. For the first, second, and third models, this process was repeated 24, 25, and 22 times, respectively, until suitable models based on significant variables and a better value of Nagelkerke’s R2 were reached. The models were built using both highly significant (*p*-value less than 0.05) and marginally significant (0.05 *p*-value less than 0.1) variables. Finally, to test the OLR assumptions, we used both proportional odds and multicollinearity for each model and variables. The proportional odds assumption means that for each variable and item that exists in the model, the ‘slope’ estimate among each pair of results across two response levels are assumed to be the same regardless of which partition we consider (The Clinical Data Experts; (Available online: https://www.quanticate.com/blog/understanding-the-proportional-odds-assumption-in-clinical-trials (accessed on 10 June 2016))). This test is an important validation step, and its rate should not be significant (less than 0.05). Multicollinearity occurs when independent variables in the model are highly associated to each other. It makes it hard to interpret the model and also creates an overfitting problem (Available online: https://towardsdatascience.com (accessed on 28 June 2022)). The obtained amount for each variable in VIF should not exceed 10.

## 3. Results

### 3.1. Descriptive Statistics

A total of 603 Tabriz residents took part in the poll. In terms of gender, women made up 59.2 percent of responses (357 individuals), while men made up 40.8 percent (246 individuals). Despite coming from a variety of age categories, the 18-year-olds were the least represented, with the bulk of respondents being between the ages of 28 and 40 at the time of the poll. The average age of respondents was 36.7 years old.

Based on Table 2, only about 10% of respondents chose the active mode as their means of commute during COVID-19 compared to around 90% who travelled by non-active modes of transport including private cars and public transport. In terms of non-commute trips, about 25% of respondents used walking and cycling as their mode choice, while around 75% declared that they opted for non-active transport. More than 17% stated that frequency of their travelling to parks per week were three times or more, whereas close to 83% travelled less than three times per week. To reach the services, 15.8% of respondents went by foot three times or more per week compared to 84.2% who walked less than three times per week.

It is apparent that the use of non-active modes for commute and non-commute purposes are considerably widespread in Tabriz, which shows the dominance of motorized travels, especially private cars. In terms of physical activity in parks, it is likely that the majority of respondents adopted the sedentary lifestyle during COVID-19.

### 3.2. Model Fit

Three OLR models were developed for this study. In the following, the ultimate models are indicated after the exclusion of insignificant factors.

#### 3.2.1. The Association between Active Lifestyle and Life Satisfaction

In this stage, after running 24 models, the best model for the identification of the correlation between active mobility and the life satisfaction was developed using the following highly significant (*p*-value of less than 0.05) and marginally significant (0.05 < *p*-value < 0.1) variables. Accordingly, apart from the control factors (age, gender, education, job, income, and commute distance) which were kept in the model, other factors including walking places2 (separation of streets from sidewalks by green spaces), perceived facilities attractiveness, population density, street length, active mobility (commute and non-commute trips), and physical activity (walking to parks and services) indicate significant relationships with life satisfaction. On this point, active mobility (commute and non-commute trip) and physical activity (walking to parks and services) have a positive and highly significant (*p*-value of less than 0.05) correlation with life satisfaction. This can explain that active lifestyle can significantly increase the levels of life satisfaction regardless of the type of physical activity. It is likely that active travel satisfaction levels have differences to the satisfaction with travelling with motorized trips. On the other hand, the high life satisfaction levels can be interpreted by relatively positive attitudes toward active transport in short trips. The most influential factor among these is the frequency of walking to the services followed by non-commute trip which show the highest amounts with estimate rates of 0.931 and 0.798, respectively. Both walking places 2 (separation of streets from sidewalks by green spaces) and facilities attractiveness show significant but weak correlations with association rates of 0.149 and 0.191, respectively. By indicating a considerable positive significance (33.432 association rate), street length demonstrates its importance in enhancing life satisfaction due to placing multiple features such as walkable sidewalks, beautiful designs, attractive monuments, pavement cafes, etc. which can hugely affect life satisfaction feelings. As a matter of fact, when the length of a street rises, the concept of the land-use mix can easily appear, provoking citizen’s positive feelings regarding their lives. The goodness-of-fit test has generated a deviance value divided by degrees of freedom proportion of 0.487 which showed a valid model because it is less than 1. The outputs of the Omnibus (“The Omnibus Test; The omnibus test is a likelihood-ratio chi-square test of the models against the null model. The *p*- value of less than 0.05 shows that the models outperform the null model (Available online: Omnibus test—IBM https://www.ibm.com/tutorials/genlin_ships_omnibus (accessed on 17 August 2021))) This test also showed validity (*p* < 0.001). The obtained rate of proportional odds for this model is 0.251 which shows good validity. In addition, the multicollinearity (VIF) test for each variable is ranged between 1.044 and 1.661 which again indicates the acceptability. The detailed results are depicted in Table 3.

#### 3.2.2. The Association between Active Lifestyle and Feeling Energetic

In this stage, after running 24 models, the optimum pattern for the recognition of the relationship between active lifestyle and the feeling of being energetic was developed using the following highly significant (*p*-value of less than 0.05) and marginally significant (0.05 < *p*-value < 0.1) variables: control factors (age, gender, education, job, income, and commute distance), walking places1 (existence of sidewalks), walking places2 (separation of streets from sidewalks by green spaces), distance to shopping malls, population density, street length, active mobility (commute and non-commute trips), and physical activity (walking to parks and services). In this regard, the classification of the independent variables for starting our first run was exactly as the variables in the first model, while the dependent variable was the second sub-criteria of subjective wellbeing, namely the feeling of being energetic. On this point, again active mobility (commute and non-commute trip) and physical activity (walking to parks and services) have a positive and highly significant (*p*-value of less than 0.05) correlation with the feeling of being energetic. It is likely that during COVID-19, using active travel modes to move toward different purposes had a prominent role in enhancing the power of mind of the individuals. Such similar trends between different travel purposes showed similar trends. Surprisingly, the pandemic-oriented restrictions and necessity of social distancing indicated many advantages to those who used walking or cycling for their journeys. In this respect, such benefits could be interpreted by a release of dopamine during physical activity which can improve mood and reduce stress. For commute travels, it is likely that respondents choose active travel for closer destinations, while by increasing the distance, the tendency to employ active modes decreases. Among these variables, commute trips along with the frequency of walking to services had the highest impacts on the feeling energetic with estimate rates of 1.348 and 1.119, respectively. Apart from walking places1 (existence of sidewalks), walking places2 (separation of streets from sidewalks by green spaces) and street length also have a positive association with the feeling of being energetic which means by increasing the existence of sidewalks, green spaces between streets and sidewalks, and street length, the feeling of being energetic also rises. This increase could also be due to the increase in walking which resulted from the growth in the above-mentioned variables. Meanwhile, the relation between the perceived distance to shopping malls and feeling of being energetic is significantly negative. This highly negative association can be interpreted to mean that as distance to shopping malls increases mentally, the likelihood of the feeling of being energetic decreases dramatically. This refers to declining the perceived energy of individuals when perceived distances of shopping centers (or job places as mentioned above) become more far, which makes the individuals less inclined to use active travel. The same results are seen for population density as well. It is likely that individuals living in densely populated areas show lesser rates of energies. The goodness-of-fit test has produced a deviance value divided by degrees of freedom proportion of 0.491 which showed a valid model. The obtained results of the Omnibus Test also showed validity for this model (*p* < 0.001). The obtained rate for proportional odds is 0.149 which shows a good validity. The multicollinearity (VIF) test for each variable is ranged between 1.060 and 1.678 indicating the acceptability of the model. The detailed results can be seen in Table 4.

#### 3.2.3. The Association between Active Lifestyle and Peaceful Mind

The ordinal logistic model for identifying the association between mobility mode choice, physical activity, and peaceful mind was completed after running 22 models, and the ultimate model was generated after omitting the non-significant indicators with higher *p*-values. The same as two previous models, we controlled some socio-economic factors (age, gender, education, job, and income) and commute distance.

As can be understood from Table 5, active mobility (commute and non-commute trip) and physical activity (walking to parks and services) have a significant positive association with a peaceful mind. As already mentioned, short trips contribute to being energetic, and as a result it is highly beneficial in reducing the stress and leading to a peaceful mind. Interestingly, the existing significance in commute trips and walking to services are considerably high which means that active commuting to job places considerably reduces the stress and revives the mind’s pain. Again, the role of dopamine cannot be ignored as a result of the impact of other factors including land-use mix, well-designed sidewalks, green spaces in the sidewalks, etc. In this manner, walking places1 (existence of sidewalks) and walking places2 (separation of streets from sidewalks by green spaces) are positively associated with the feeling of being relaxed/peaceful. It is likely that areas with sufficient green spaces and pleasant designs are more rewarding in having positive contribution to a peaceful mind; however, these associations are weak. Conversely, significant negative correlation was seen between perceived distance to the park and the feeling of being relaxed/peaceful. As elaborated in previous models, as the distance to some areas such as parks rises, the likelihood of feeling of being energetic decreases intensely. This negative association is similarly seen for population density. Table 5 represents the details of our model in terms of the association between active lifestyle and being relaxed/peaceful. The same as two previous models, the goodness-of-fit test has indicated a deviance value divided by degrees of freedom proportion of 0.488 which expressed a valid model. The obtained results of the Omnibus Test also indicated validity for this model. The amount for proportional odds is 0.196 which indicates good validity. Moreover, the multicollinearity (VIF) test for each variable is ranged from 1.036 to 1.672 which again indicates the acceptability.

All things considered, the existing models demonstrated similar patterns despite having minor differences which indicate that active mobility in all modes have positive impacts on the subjective wellbeing of individuals during COVID-19. In this regard, the role of urban features such as green spaces and sidewalk designs are directly and indirectly promising in the subjective wellbeing of citizens. In addition, in the COVID-19 era, it is likely that individuals inevitably are inclined to use active types of travel for short distances.

## 4. Discussion

The current study analyzes the associations among different modes of active lifestyle (including job commute, non-commute, commute to services, and commute to parks) and wellbeing (including life satisfaction, being energetic, and peaceful mind) during the COVID-19 pandemic in Tabriz. In this paper, we controlled both socio-economic and commute distance variables. Along with the mentioned variables used in this study, we utilized some relevant objective and subjective variables to enhance and enrich the model fit. We developed three separate models considering the number of dependent variables for this aim. In each model, we incorporated any above-mentioned types of active transport simultaneously to explore the associations.

A large body of literature emphasizes the association of non-commute walking with SWB [1,57]. Similarly, the condition is somehow the same for active commuting to a job place [58]. The large volume of these studies have been carried out before the emergence of COVID-19, while there is less evidence on the association between active transport and SWB during the pandemic [59,60,61]. These research studies have chiefly been conducted in the fields of medicine, psychology, and public health. However, the role of objective and subjective variables of the built environment has mainly been glossed over. Less studies address the association between active mobility and SWB, emphasizing the geographical and environmental characteristics [62,63]. Moreover, there is less evidence of taking a holistic approach to the correlation of various active mobility modes with wellbeing domains by practitioners or scholars. Furthermore, the majority of these studies have been accomplished in developed countries [64,65]. All we know is that this is the first interdisciplinary analysis that comprehensively (holistically) investigates the correlations among active travel, socio-economic and physical commute distance (controlling factors), objective and subjective characteristics of built-environment, and subjective wellbeing either before or during the COVID-19 pandemic in a developing country. Our findings driven by ordinal logistic regression demonstrate the existence of correlations in the impact of different modes of active travel on three domains of subjective wellbeing. The models indicated high similarities in the correlational significance of four types of active travel with any kind of wellbeing. However, some differences existed in the direction (positive or negative) of significant variables.

In our first model which explored the associations between active mobility (commute ^1^ and non-commute ^2^), physical activity (walking frequencies to parks ^3^ and services ^4^ per week), and life satisfaction, any of the four mentioned variables indicated significant correlations with life satisfaction while controlling for factors including commute distance and individual-level factors such as age, gender, education, job, and income. The overall outcome is in line with some studies before [15,18,20,66,67] and during COVID-19 [61,68]. However, in terms of active commute travel, this correlation is marginal, which can be related to the dominance of private cars in commuting trips during the pandemic. Furthermore, due to the cultural, economic, local planning strategies, and finally, the structure of the cities of Iran, having and using private cars are considered invaluable, which considerably encourages the individuals to own and utilize that. According to Kamelifar et al. (2022), due to pandemic anxiety, the use of public modes of transport such as bus transit and taxi considerably have declined in Tabriz during COVID-19. It is likely that the most of commuters do not use public transport during the pandemic or work remotely. All of these outcomes show the strong impact of the COVID-19 mediator. These facts are essential for interpreting results during COVID-19. In terms of the other significant objective and subjective variables, the finding showed that the factor of “separation of street from sidewalk by green spaces” [56]. It is likely whether the majority of commuters do not use public transport during the pandemic or work remotely. All of these outcomes show the strong impact of the COVID-19 mediator. These facts are important for interpreting results during COVID-19. In terms of other significant objective and subjective variables, the finding showed that the factor of “separation of street from sidewalk by green spaces” [69,70], perceived attractiveness of facilities [71,72,73], and street length (in line with Molaei et al., 2021) [74] indicated positive associations with life satisfaction during COVID-19. The factor of street length indirectly affects the life satisfaction and wellbeing of adults by enhancing their walking desire. For example, increased accessibility (from abundant streets) to a wide range of facilities/services is key during pandemic crises [75] as it allows participation in activities and access to a variety of local services/facilities. It allows participation in activities and access to various local services/facilities. Furthermore, while a few studies confirm the positive correlations between population density and life satisfaction due to reduced urban mobility during COVID-19 [66,67,76], the obtained results of this study as well as a majority of conducted research studies showed negative associations with wellbeing and life satisfaction which is consistent with the study of Mouratidis (2022) [77]. This can be interpreted that in our case, the residents of denser neighborhoods typically live in smaller dwellings, rely more on public transport, and have lower access to green space.

Our second model explored the connections between four types of commute and physical activity (commute, non-commute, physical activity frequencies to park, and services per week) and the subjective factor of feeling energetic. Similar to the first model’s pattern, any of the four active travel types expressed significant associations with the characteristic of feeling energetic. However, these correlations are strong. This result is in line with these studies before and during COVID-19 [78,79,80,81,82]. Chng et al. (2016) found that commute trip is associated with higher rates of liveliness and being energetic in London (after controlling for commute distance), showing that active travel to work may be advantageous in large cities [40]. However, in some cases, active commuting to the job is associated with lower energetic feelings [83]. Based on the finding of this article, by increasing the frequency in the volume of physical activity in parks and services, adults feel more energetic during the pandemic. It means those who experience physical activity (e.g., jogging, walking, cycling, etc.) more than three times per week have better mental health. In this model, the objective factors, including street length (positively) and population density (negatively), indicated significant correlations with the factor of feeling energetic. As the length of the streets increases, so does the length of sidewalks, resulting in better conditions for walking in the neighborhood. However, it has been said that these sites for walking preferably should be accompanied by green spaces and trees along the path [84,85]. In terms of the factor of population density, congested areas decrease the willingness to exercise and engage in physical activity compared to the least densely populated areas. In other words, living in higher populated areas declines the feeling of happiness, feeling energetic, and subjective wellbeing resulting from lower physical activities. These results are aligned with the studies of [25,86]. In our case study, this can also be interpreted that due to the pandemic anxiety, the sedentary lifestyle has increased in dense districts during COVID-19. In parallel, both subjective factors of perceived existence of sidewalks and separation of the street from the sidewalk by green spaces were positively correlated with feeling energetic, indicating the consistency of both objective and subjective factors’ results in this model. Apart from it, the perceived distance to shopping malls was negatively associated with feeling energetic. This means that residents prefer closer shopping. This finding calls for the role of mixed use in raising the energetic feeling and subjective wellbeing, which is in line with studies [87,88,89]. Latest studies have consistently shown that spaces with high mixed uses are related to a greater level of perceived social support and higher levels of being mentally energetic [90,91]. A mix of uses can encourage social interaction as individuals meet at local facilities such as shops and pubs. They provide destinations for citizens to walk to in the neighborhood and sources for physical activity, which indirectly increases the perceived feeling of energy among adults.

Our third and final model addressed the associations between different types of physical activity (commute, non-commute, frequency of physical activity to parks, and services per week) and the subjective factor of peaceful mind (as the third component of wellbeing in this study). Like the two former models, all active modes of travel were correlated with peaceful mind (consistent with the studies of Bird et al., Coughenour et al., Viana & de Lira, and Schuch et al.) [12,92,93,94]. Of the objective variables, only the factor of population density was negatively correlated with peaceful mind which indicates that adults living in low density areas are more willing to take part in active living as elaborated in the former paragraphs. Similar to our second model, two variables of existence of sidewalks, separation of street from sidewalk by green spaces and (positively) and perceived distance to offices (negatively), were associated with peaceful mind.

Overall, we found that active commute, non-commute, and frequency of physical activity in parks and services have significant positive impacts on subjective wellbeing dimensions, including life satisfaction, feeling energetic, and a peaceful mind. Hence, these findings indicate that staying physically active over the pandemic is important, regardless of the physical activity domain. However, this effect differs following the various types of physical activity. Accordingly, different patterns have appeared. In other words, multiple types of physical activity have different estimations and predictions on the variety of self-assessed mental wellbeing in Tabriz, an indicator of a developing country’s metropolitan area. The finding also supports the debate that active commuters and non-active individuals have taken different lifestyles and environments, and thus, various factors must be controlled to avoid biased results [95]. Generally, interconnections among the activity measures are positive and have a slight to moderate size. It represents that individuals who are active in one activity indicator (e.g., walking or cycling) are probably active in several areas of activity (e.g., biking to work). This finding suggests some central implications. Although we cannot list the advantages of walking for different commute and activity purposes on different types of mental wellbeing solely based on these results, the subjective benefits of active lifestyle mainly come from active living. This suggests that decision-makers, stakeholders, and planners should take the diverse interventions into consideration to encourage individuals to take active lifestyle within, to, and from their built environment. For example, urban organizations can build attractive pavements and other areas for promoting physical activity. These authorities can suggest mixed-use patterns where dwellers can have accessibility to various destinations within short distance and include walking in their daily life principles. Including green spaces, benches, shade, and leisure facilities could increase all age groups’ convenience and aesthetic experience. This implication is in line with that of other studies encouraging the integration of mental health issues, land use, and active transportation systems to pull residents out of their sedentary living habits in all periods, including before and during COVID-19 [56,96]. Apart from the suggested environmental justifications, increased awareness and knowledge regarding active transportation’s environmental and health benefits may be important in promoting active transportation.

## 5. Strengths and Limitations

The timeline of the survey during the COVID-19 period, the collection of retrospective data to capture the period during the pandemic, and the use of standardized self-reported subjective wellbeing measures were all strengths of this study, in addition to the use of different types of active lifestyle targets in subjective wellbeing. Some limits, however, were mentioned. The information was acquired during the pandemic, which necessitated an internet sampling of 603 respondents. With a larger sample size, more credible data for modeling the relationships between various forms of active lifestyles and subjective wellbeing might be collected. Young adults from middle-income families made up the majority of the sample. In addition, in this study, we focused on Iran citizens, and it might be biased to generalize the outputs for the whole cities located in MENA region; however, the results can be an indicator of large cities of MENA due to the similarities which were mentioned already.

## 6. Conclusions

The current research aimed to identify the associations between different types of the active lifestyle (including active commute, active non-commute, and frequency of active trips to parks and services per week), subjective wellbeing (including life satisfaction, feeling energetic, and peaceful mind) during the COVID-19 pandemic. It was carried out among the adults in one of Iran’s metropolitan areas (Tabriz, Iran) as an example of a developing country site. To conclude, multiple lessons are obvious from this research. In closing, our results further support the importance of engaging in an active lifestyle, as this is correlated with maintaining subjective wellbeing.

First, the volume of studies evaluating the mentioned relationship is considerably rare in the universal context. These studies predominantly have been conducted in developed countries of North America and Europe, limiting our findings generalization to other contexts [97]. Such scarcity is even more during COVID-19. Moreover, the large volume of these studies has been conducted without incorporating objective (street length, land-use mix, density etc.) and subjective variables (perceived walkable environment, perceived distance to services, the perceived attractiveness of facilities etc.) of the built environment. Accordingly, this is one of the first steps accomplished in MENA countries during COVID-19.

Second, although based on the literature and finding of this study there is a positive association between increased physical activity/active lifestyle and improved subjective wellbeing, further longitudinal studies in city or neighborhood scales are needed to achieve more accurate, reliable, and robust findings. This need is also more demanded during COVID-19. The impact of the COVID-19 outbreak on subjective wellbeing may be continuous and long-term [98,99]. Thus, urban authorities along the public health agencies must offer and implement appropriate and effective interventions, in which active lifestyle should be a priority and preferred action.

Third, despite achieving similar results which conformed to the significant and positive impact of different active lifestyles on subjective wellbeing, the estimation patterns are different in the provided models. For example, this amount in commute trips ranges from 0.480 in life satisfaction to 1.348 in feeling energetic. Thus, a unit increase in active lifestyles would lead to different raise outcomes in the dependent variable of subjective wellbeing, which should be considered in planning and policymaking.

Fourth, besides the existence of significant relationships between various kinds of active lifestyles and different variables of subjective wellbeing, the rest of the significant indicators (without taking into consideration the controlling factors) which affect subjective wellbeing are:

- First model: perceived separation of the street from the sidewalk by green spaces, perceived facilities attractiveness, population density, and street length.
- Second model: perceived existence of sidewalks, perceived separation of street from the sidewalk by green spaces, perceived distance to shopping malls, population density, and street length.
- Third model: perceived existence of sidewalks, perceived separation of the street from the sidewalk by green spaces, perceived distance to parks, and population density.

Overall, the obtained results from this study holistically add new evidence to the existing COVID-19 literature and have made an important contribution to enabling subjective wellbeing, with an outcome of various kinds of activities and neighborhoods that have been largely neglected. In addition, the obtained results provide a chance to suggest some intervention policies and implications to similar towns in MENA or other developing nations to encourage individuals toward the active lifestyle. Consequently, there is a significant requirement for a more integrated COVID-19 study in all nations and countries that enables scientists and academicians to analyze and help stakeholders and policymakers have accurate plans to spread out healthy communities.

## Figures and Tables

**Table 1:** Socio-economic, subjective, and objective variables.

Variables	Description and Coding
Age	Continuous
Gender	Female = 1, Male = 0
Education	Diploma and Undergraduate = 1, Bachelor = 2, Master = 3, PhD and higher = 4
Income	Continuous
Job	Unemployment = 1, Housewife = 2, Student = 3,Employee = 4 Freelance = 5, Retired = 6
P. Distance to Different Services/Land uses	Less than 5 min = 1, 5 to 10 min = 2, 10 to 20 min = 3, 20 to 30 min = 4, More than 30 min = 5
Perceptive Walkable Places (1. Existence of sidewalks, 2. Separation of street from sidewalk by green spaces, 3. Existence of shortcut routs)	From very little = 1 to very much = 5
Facilities Attractiveness	No existence = 1, Not attractive at all = 2, Not very attractive = 3, Medium = 4, Acceptable Attractiveness = 5, Very attractive = 6
Objective Built Environment (Commute distance- Street length, Land use mix, Number of Intersections, Building and Population density)	Continuous
Mobility Mode Choice (commute & non-commute)	Active (Walking & Cycling) = 1, Other modes (e.g., Private car, Public transport, Metro and …) = 0
Frequency of Walking to Parks/Services	Three or more than three times per week = 1, Less than three times per week = 0
Subjective Wellbeing (1. Feeling of life satisfaction, 2. Feeling of Being Energetic, 3. Feeling of being relaxed/peaceful)	From Very Low = 1 to Very High= 10

Source: author elaboration.

**Table 2:** Active commute and non-commute trips and frequency of walking to parks and services.

Category		Active	Non-Active	Category		More than 3 Times Per Week (Active)	Less than 3 Times a Week (Non-Active)
Commute trips	Frequency	62	535	Walking to parks	Frequency	107	496
	Percent	10.3%	88.7%		Percent	17.7%	82.3%
Non-commute trips	Frequency	147	454	Walking to services	Frequency	95	508
	Percent	24.4%	75.3%		Percent	15.8%	84.2%

Source: SPSS software calculations.

**Table 3:** Model for the association between active lifestyle and life satisfaction.

Variable/Measure	Estimate	Std. Error	Wald	df	*p*-Value	95% Confidence Interval	
						Lower Bound	Upper Bound
Age	0.004	0.011	0.103	1	0.748	−0.018	0.025
Gender	0.156	0.208	0.566	1	0.452	−0.251	0.564
Education	−0.098	0.152	0.412	1	0.521	−0.397	0.201
Job	0.283	0.159	3.177	1	0.075	−0.028	0.593
Income	0.008	0.009	0.831	1	0.362	−0.009	0.025
Commute distance	−0.087	0.079	1.219	1	0.269	−0.241	0.067
WalkingPlaces2(Separation of street from sidewalk by green spaces)	0.149	0.082	3.326	1	0.068	−0.011	0.309
Facilities attractiveness	0.191	0.082	5.469	1	0.019	0.031	0.350
Population density	−0.153	0.086	3.148	1	0.076	−0.322	0.016
Street Length	33.432	11.68	8.182	1	0.004	10.524	56.340
Commute trip	0.480	0.246	3.789	1	0.052	0.003	0.962
Non-commute trip	0.798	0.398	4.010	1	0.045	0.017	1.579
Walking to green spaces	0.620	0.310	3.996	1	0.046	0.012	1.228
Walking to services	0.931	0.313	8.845	1	0.003	0.317	1.544
**Model Summary**	**−2 Log likelihood**		**Chi-Square**	**df**	***p*** **-value**	**Nagelkerke R^2^**	
	1433.000		50.986	14	<0.0001	0.245	
**Goodness of fit**			**Omnibus Test**				
	**Value**	**df**	**Value/df**		**Likelihood** **Ratio X^2^**	**df**	***p***-**value**
**Deviance**	1515.624	3109	0.487		52,988	14	<0.0001
**Pearson Chi-Square**	3059.308	3109	0.984				
**Log Likelihood**	−757.812						

Source: SPSS software calculations.

**Table 4:** Model for the relationship between active lifestyle and feeling energetic.

Variable/Measure	Estimate	Std. Error	Wald	df	*p*-Value	95% Confidence Interval	
						Lower Bound	Upper Bound
Age	−0.021	0.011	3.574	1	0.059	−0.042	0.001
Gender	−0.259	0.207	1.555	1	0.212	−0.665	0.148
Education	−0.185	0.150	1.522	1	0.217	−0.479	0.109
Job	0.246	0.158	2.430	1	0.119	−0.063	0.556
Income	0.047	0.082	0.326	1	0.568	−0.207	0.114
Commute distance	0.000	0.009	0.001	1	0.979	−0.017	0.017
WalkingPlaces1 (Existence of sidewalks)	0.188	0.082	5.307	1	0.021	0.028	0.348
WalkingPlaces2 (Separation of street from sidewalk by green spaces)	0.149	0.082	3.326	1	0.068	−0.011	0.309
p. Distance to shopping malls	−0.325	0.093	12.274	1	0.000	−0.508	−0.143
Population density	−0.153	0.086	3.148	1	0.076	−0.322	0.016
Street Length	33.432	11.68	8.182	1	0.004	10.524	56.340
Commute trip	1.348	0.401	11.317	1	0.001	0.563	2.133
Non-commute trip	0.681	0.249	7.501	1	0.006	0.194	1.168
Walking to green spaces	0.813	0.308	6.962	1	0.008	0.209	1.417
Walking to services	1.119	0.315	12.656	1	0.000	0.503	1.736
**Model Summary**	**−2 Log likelihood**		**Chi-Square**	**df**	***p*** **-value**	**Nagelkerke R^2^**	
	1422.909		76.294	15	<0.0001	0.208	
**Goodness of fit**				**Omnibus Test**			
	**Value**	**df**	**Value/df**		**Likelihood Ratio X^2^**	**df**	***p*** **-value**
**Deviance**	1424.469	2901	0.491		44.392	15	<0.0001
**Pearson Chi-Square**	1390.606	2901	0.994				
**Log Likelihood**	−712.235						

Source: SPSS software calculations.

**Table 5:** Model for the association between active lifestyle and peaceful mind.

Variable/Measure	Estimate	Std. Error	Wald	df	*p*-Value	95% Confidence Interval	
						Lower Bound	Upper Bound
Age	−0.020	0.011	3.390	1	0.066	−0.042	0.001
Gender	−0.283	0.203	1.942	1	0.163	−0.682	0.115
Education	−0.153	0.148	1.057	1	0.304	−0.444	0.138
Job	0.183	0.159	1.333	1	0.248	−0.128	0.495
Income	0.085	0.081	1.087	1	0.297	0.074	0.244
Commute distance	−0.004	0.009	.191	1	0.662	−0.021	0.013
WalkingPlaces1 (Existence of sidewalks)	0.215	0.090	5.735	1	0.017	0.039	0.391
WalkingPlaces2 (Separation of street from sidewalk by green spaces)	0.183	0.082	5.003	1	0.025	0.023	0.343
*p*. Distance to park	−0.285	0.091	9.822	1	0.002	−0.464	−0.107
Population density	−0.156	0.083	3.575	1	0.059	−0.318	0.006
Commute trip	0.971	0.399	5.923	1	0.015	0.189	1.752
Non-commute trip	0.649	0.247	6.891	1	0.009	0.164	1.133
Walking to green spaces	0.765	0.304	6.348	1	0.012	0.170	1.360
Walking to services	1.050	0.315	11.133	1	0.001	0.433	1.666
**Model Summary**	**−2 Log likelihood**		**Chi-Square**	**df**	***p*** **-value**	**Nagelkerke R^2^**	
	1449.137		65.846	14	<0.0001	0.180	
**Goodness of fit**			**Omnibus Test**				
	**Value**	**df**	**Value/df**		**Likelihood** **Ratio X^2^**	**df**	***p*** **-value**
**Deviance**	1437.015	2947	0.488		53.054	14	<0.0001
**Pearson Chi-Square**	2944.898	2947	0.999				
**Log Likelihood**	−718.507						

Source: SPSS software calculations.

## Data Availability

At the time of the publication of this paper, the data have not been shared on a repository.
